# Deferent Anatomical Presentations of Iliolumbar Ligament: A Cadaveric Study

**DOI:** 10.1155/2022/5992510

**Published:** 2022-11-02

**Authors:** Abdulaziz Saleh Albatati, Amar Fathi Mohamed Khalifa, Mohamed El-Sherbiny, Musaad Abdulaziz Alfayez, Saeed Abualmakarim, Hasnaa Ali Ebrahim, Anan Rajeh Aljahdali, Hasim Darwish

**Affiliations:** ^1^Department of Basic Medical Sciences, College of Medicine, AlMaarefa University, P.O. Box 71666, Riyadh 11597, Saudi Arabia; ^2^Department of Anatomy, Faculty of Medicine, Mansoura University, Mansoura, Egypt; ^3^Department of Anatomy, College of Medicine, King Saud University, Saudi Arabia; ^4^Department of Basic Medical Sciences, College of Medicine, Princess Nourah bint Abdulrahman University, P.O. Box 84428, Riyadh 11671, Saudi Arabia; ^5^Biological Sciences Department, Faculty of Science, University of Jeddah, Saudi Arabia

## Abstract

This work was carried out to describe the detailed gross anatomy of the iliolumbar ligaments in human cadavers and to shed more light on these disputes regarding the configuration and direction of these ligaments. Twenty partially dissected human formalin-preserved cadavers originating from North America and Europe were investigated in this study. Blunt dissection was made through the ventral and dorsal aspects of the pelvic area of the cadavers. According to the current study, the anterior and posterior portions of the iliolumbar ligament most frequently attached to the 5^th^ lumbar vertebra's transverse process (70% and 80%, respectively). The body of the 4^th^ lumbar vertebra with the 5^th^ lumbar vertebra' transverse process was the attachment of the anterior part (30%). The attachment of the posterior part was the body of the 5th lumbar vertebra (20%). The anterior and posterior parts of the iliolumbar ligament were inserted into the anterior tip of the iliac crest. There is an obvious variation in the morphological appearance of the iliolumbar ligament distinguished in attachments, length, width, thickness, number of bands, and the presence of accessory bands in the anterior part of the ligament. In addition, a new attachment for the anterior band was revealed in one-third of the specimens (body of the 4th lumbar vertebra) which have not been described before. Also, in one-fifth of the specimens, there was a new attachment for the posterior band (body of the 5th lumbar vertebra).

## 1. Background

The axial skeleton and pelvis are attached by three vertebropelvic ligaments. The iliolumbar (IL), sacrotuberous, and sacrospinous ligaments are among them. These ligaments link numerous pelvic tissues from the interior and exterior aspects. Additionally, they offer essential structural support for these components [1].

A great fan-shaped, composite ligament outspreads laterally from the lower anterior portion and the tip of the fifth lumbar vertebra (L5) transverse process to get inserted into the top of the iliac crest and sacroiliac joint capsule by being directed posterolateral [2–4].

**Figure 1:**
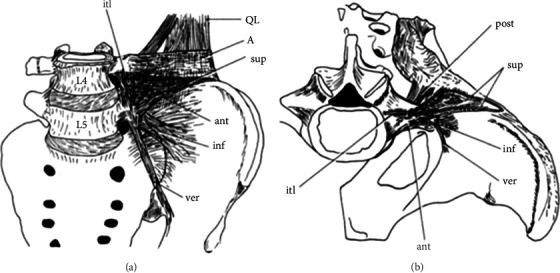
Parts of the iliolumbar ligament [5]: (a) front view. (b) top view. A: anterior layer of the thoracolumbar fascia; QL: quadratus lumborum; sup: superior portion of the IL ligament; ant, anterior portion of the IL ligament; inf, inferior portion of the IL ligament; post: posterior portion of the IL ligament; ver: vertical portion of the IL ligament; itl: intertransverse ligament.

Routine autopsies and anatomical dissections revealed marked inconsistencies concerning description of IL parts highlighting the variability in number and form. Some studies reported five parts of IL, which included anterior, posterior, superior, inferior, and vertical iliolumbar divisions as shown in (Figure 1). The whole anteroinferior border of the L5 transverse process is covered by a well-developed ligamentous band that constitute the anterior portion of the iliolumbar ligament, while the anterior and posterior thickenings of the fascia that enclose the quadratus lumborum muscle base are assumed to constitute the superior portion. The portion, which reaches the ligamentous region of the ilium behind the origin of the quadratus lumborum from the tip and posterior border of the L5 transverse process, is the posterior one. The lower border of the L5 transverse process and the body of LS represent the origin of the inferior part, which runs downwards and laterally over the surface of the anterior sacroiliac ligament to get attached into the upper and posterior parts of the iliac fossa. The vertical portion of the iliolumbar ligament spreads from the anteroinferior border of the L5 transverse process to get inserted into the posterior end of the iliopectineal line of the pelvis in a vertical direction [3, 5]. A study by [6] using 100 specimens revealed the presence of only two anterior and posterior parts constituting the ligament. Moreover, this study proved that both parts extend between the L5 transverse process and the iliac crest in a posterolateral direction.

An earlier study has described the presence of the quadratus lumborum muscle sandwiched between the anterior and posterior portions of the IL ligament [7].

A study carried on cadavers revealed the morphological correlation between the L5 transverse process size and the iliolumbar ligament width showing that a wider transverse process of L5 is related to broader posterior portion of the iliolumbar ligament [8]. The purpose of this study is to explore the precise gross anatomy of the IL ligament in human cadavers as a part of the vertebropelvic ligament complex shedding more light on these disputes regarding the configuration and direction of its anterior and posterior divisions, which according to many studies represent the main bands that play a vital role in the biomechanical properties of the lumbosacral junction [9, 10].

## 2. Material and Methods

### 2.1. Anatomical Study

Twenty partially dissected human formalin-preserved cadavers that originated from North America and Europe, with age ranging from 55 to 85 years old (according to the referred file of each cadaver), were investigated in this study. They were obtained from the dissecting room of the anatomy department at the faculty of Medicine, King Saud University, Riyadh district. The causes of death in these cadavers were usually attributed to medical etiologies, and the gross examination showed no obvious low back deformity.

To approach the ligaments, blunt dissection was made through the ventral and dorsal aspects of the pelvic area of the cadavers. All muscles and internal organs were removed, whereas all ligamentous structures were preserved.

At the time of dissection, many of these specimens were partially dissected before. For exposing the ligamentous structure, the remaining parts of the skin, the superficial fascia, and the deep fascia were removed for all specimens. For the anterior bands, the peritoneal viscera were removed also with the psoas major, psoas minor (if present), and quadratus lumborum muscles in all specimens. Any deep fascia, vessels, or nerves were also removed. Unfortunately, genitalia were removed before, so we could not distinguish the gender for these cadavers clearly.

Furthermore, the iliolumbar ligament's anterior band was visible on the anterior aspect of the specimens at the start of our study showing the oblique extension of the IL ligament from the fourth and fifth lumbar vertebrae to its insertion into the anterior or the anterior-medial margins of the iliac crest.

On the other hand, for exposing the posterior band, muscles of the back with the gluteus muscle group were removed showing the iliolumbar ligament's portion extending from the fifth lumbar vertebra to get inserted into the posterior aspect of the iliac crest.

A digital Vernier caliper was used to measure the length, width, and thickness of the ligaments in situ after determining the above observations. The length of iliolumbar ligament bands was measured from the tip of the L5 transverse process to the attachment at the iliac crest, while the width and thickness were taken at the middle part of the ligament for only the anterior band as previously described in [10, 11].

## 3. Results

The frequency of origins, insertions, number of bands, and accessory bands of the iliolumbar ligament are seen in Table 1. It was found that the most frequent origin of the anterior and posterior parts of the iliolumbar ligament is the 5^th^ lumbar vertebra's transverse process (TP) (70% and 80%, respectively), while in 6 (30%) specimens, the anterior part was originating from the 4th lumbar vertebra's body with the 5th lumbar vertebra's TP. In a lower number of specimens 4 (20%), the origin of the posterior part was the 5th lumbar vertebra's body. All of the specimens revealed that the iliolumbar ligament's anterior and posterior parts were inserted into the iliac crest's anterior tip.

However, the accessory band was described as any band originating from the same origin as the main ligament but it is either inserted at different sites or totally separated from the main band.

Furthermore, the most frequent number of bands of the anterior part was 3 and 5 (70%). In contrast, the posterior part showed mainly one band (95%). Concerning the frequency of accessory bands, the anterior part exhibited mostly (69.2%) one band, while in 7 (35%) specimens, the posterior part had accessory bands that were fused with the opposite anterior ones.

We found in all specimens that the length of the iliolumbar ligament's anterior band ranged from 1.22 cm to 5.9 cm with an average of 4.06 ± 1.45 cm. On the other hand, the posterior part was shorter with an average length of 2.44 ± 0.88 cm. As regards the width of the anterior band, it ranged from 0.62 cm to 2.60 cm with a mean of 1.34 ± 0.54 cm, while the thickness of this band was 0.13-0.78 cm with a mean of 0.41 ± 0.21. Moreover, the anterior part showed 1-6 bands, while the posterior part had only 1-2 bands (Table 2).

**Figure 2:**
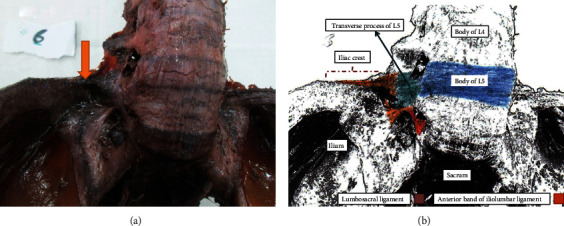
(a, b) Show the right iliolumbar ligament (anterior view).

Figure 2 illustrates the right anterior iliolumbar ligament. The transverse process of L5 was the origin of both anterior and posterior bands, which were both inserted in the iliac crest. The anterior band measured 0.62 cm in thickness, 1.22 cm in length, and 62 cm in width. The anterior band exhibited 5 bands with an extra 2 accessory band. Alternatively, the posterior band showed a length of 1.14 cm with only one band (Figure 2).

**Figure 3:**
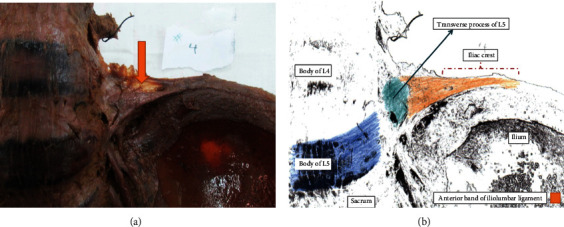
(a, b) Show the left iliolumbar ligament (anterior view).

Figure 3 illustrates the left iliolumbar ligament. The origin of the anterior band was the body of L4 and transverse process of L5, while the posterior band origin was the transverse process of L5. Both bands were inserted in the iliac crest. The anterior band measured 0.27 cm in thickness, 5.69 cm in length, and 2.6 cm width. The anterior band exhibited 5 bands with an added 2 accessory bands. Alternatively, the posterior band showed a length of 2.34 cm with only one band (Figure 3).

**Figure 4:**
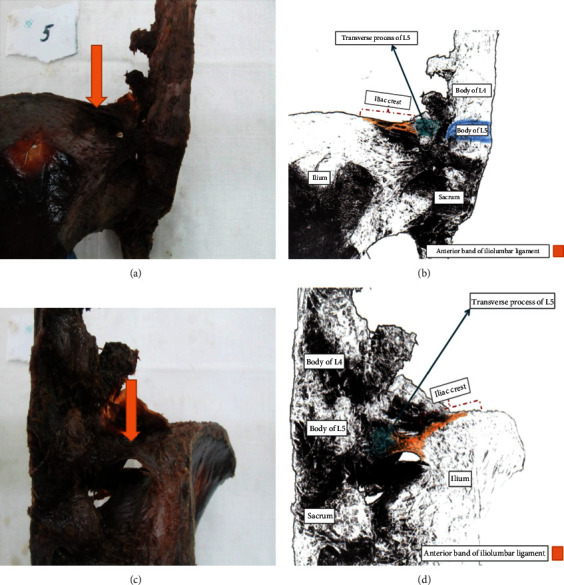
The right iliolumbar ligament (anterior view (a, b); posterior view (c, d)).

Figure 4 illustrates the right iliolumbar ligament. The origin of the anterior and posterior bands was the transverse process of L5, and both were inserted in the iliac crest. The anterior band measured 0.29 cm in thickness, 4.48 cm in length, and 1.37 cm in width. The anterior band exhibited 5 bands with an added 1 accessory band. Alternatively, the posterior band showed a length of 2.24 cm with only one band (Figure 4).

## 4. Discussion

This morphometric study revealed that the IL ligament had two anterior and posterior bands. Moreover, the anterior band showed several bands ranging from 1 to 6, while the posterior one displayed mainly (95%) one band (Table 2). Concerning the frequency of accessory bands, the anterior part exhibited mostly (69.2%) one band, while in 7 (35%) specimens, the posterior part displayed accessory bands that were fused with the opposite anterior ones (Table 1).

Routine autopsy and anatomical dissections really showed disagreements about the configuration of the IL ligament. According to reports, the IL ligament is divided into five parts: the anterior, posterior, superior, inferior, and vertical [3]. However, a prior investigation using 100 specimens found that the ligament only had two anterior and posterior sections [6]. Later, computer-assisted reconstruction of the IL ligament served to confirm this [12]. However, an extra lumbosacral band and a sacroiliac portion have also been reported [2].

Black and white persons have morphologic variations in the IL ligament, according to Hanson and his colleagues. In contrast to white people, who have two shorter bands, black people have a single, noticeably longer band that makes up the ligament. Additionally, they noted that the IL ligament was positioned more horizontally in black individuals compared to white individuals [13]. Moreover, Fujiwara et al. conducted research on the morphology of the iliolumbar ligament in 56 human cadaver lumbosacral spines that had been embalmed. In 74 ligaments, anterior and posterior portions displayed different courses, whereas in 32 ligaments, these portions moved in unison. They also claimed that compared to female anatomic specimens, the posterior iliolumbar ligament in men was noticeably shorter and directed more posteriorly [11].

The transverse process (TP) of the fifth lumbar vertebra was the most frequent origin of the anterior and posterior portions of the iliolumbar ligament (70% and 80%, respectively), in the current study (Figures 2 and 4) (Table 1). In 6 (30%) specimens, the origin of the IL ligament's anterior portion was from the body of the 4th lumbar vertebra with the TP of the fifth lumbar vertebra (Figure 3) (Table 1). The body of the fifth lumbar vertebra served as the origin of the IL ligament's posterior portion in fewer number of specimens 4 (20%) (Table 1). Regarding the insertion, all specimens showed that the IL ligament's anterior and posterior portions were attached to the anterior tip of the iliac crest (Figures 2–4).

100 specimens were examined by Hanson and Sonesson, who established that the anterior and posterior portions both arise from the fifth lumbar transverse process and extend laterally and posteriorly to the iliac crest [6]. Similar to this, Pool-Goudzwaard and his colleagues found that the IL ligament mostly originates from the tip and lower anterior portion of the fifth lumbar vertebra, with IL sometimes having a weak connection to the transverse process of the fourth lumbar vertebra. The ligament then reaches the top of the iliac crest and the sacroiliac joint capsule by running posterolateral [2].

The anterior band of the iliolumbar ligament, which is broad and flat, originates from the anteroinferior and lateral parts of the fifth lumbar transverse process and expands as a wide fan before inserting on the anterior part of the iliac tuberosity beneath the posterior band, according to a study of 28 iliolumbar ligaments performed on 14 adult volunteers using magnetic resonance imaging. The posterior band, on the other hand, inserts on the iliac crest and originates from the tip of the fifth lumbar transverse process being thinner and more rounded (from the anterior margin to the apex) than the anterior band [14]. Likewise, a recent study reviewing 100 whole-body PECT-CT of adult male and female patients confirmed that lumbar 5 is the main origin of the IL ligament [15].

Analysis of the IL measurements revealed that the length of the iliolumbar ligament's anterior band ranged from 1.22 cm to 5.9 cm with an average of 4.06 ± 1.45 cm. On the other hand, the posterior part was shorter with an average length of 2.44 ± 0.88 cm. As regards the width of the anterior band, it varied from 0.62 cm to 2.60 cm with an average of 1.34 ± 0.54 cm, while the thickness of this band was 0.13 cm to 0.78 cm with an average of 0.41 ± 0.21. The iliolumbar ligament's anterior band was previously measured by Hanson and Sonesson [6], who observed that it varied in length between 30 and 40 mm, in width from 8 to 10 mm, and in thickness from 2 to 3 mm, whereas measurement of the iliolumbar ligament's posterior band varied in length between 10 and 12 mm and 5 to 7 mm. In a study on imaging done by Rucco et al., measurement of the anterior band revealed that it varied from 10 to 15 mm long and 2 to 3 mm wide and the posterior band was 15 to 20 mm in length and 1 to 3 mm in width [16]. According to Hanson et al., the average length of the IL ligament in Caucasians is about 33.2 mm, whereas it is 61.88 mm in African Americans [13], while in a different imaging research, the anterior iliolumbar ligaments varied from 14 to 34 mm (average, 24 mm) in length, and the posterior iliolumbar ligament's varied from 9 to 26 mm (average, 17 mm) [10].

In addition, a more recent study used frozen sections taken from 29 people, where length, surface, volume, and angle of positional relations were assessed using computers, and 7-tesla magnetic resonance images were created to differentiate the anterior and posterior components of the IL ligament. Virtual reconstructions showed that the anterior and posterior portions, respectively, were 30 and 25 mm in length, 17 to 19 mm in height, and 4 mm thick [12].

## 5. Conclusion

There is an obvious variation in the morphological appearance of the vertebra-pelvic ligament complex distinguished in the iliolumbar ligament, the sacrospinous ligament, and the sacrotuberous ligament.

This variation includes the origin, insertion, length, width, thickness, number of bands, and the presence of accessory bands in the iliolumbar ligament's anterior band, but it includes the origin, length, and number of bands for the iliolumbar ligament' posterior band.

The sacrospinous and the sacrotuberous ligaments, on the contrary, are different only in terms of their length, width, and thickness.

This morphological variation may cause instability to the sacroiliac joint and could be related to unexplained lower back pain, which may correlate with some of this variant morphological appearance more than others.

Although, a few earlier studies showed that the occurrence of disc prolapse in the region of fourth-fifth lumbar vertebrae may be correlated with the strength and direction of the iliolumbar ligament, we suggest that it could also be related to the morphological variation found in our study.

## 6. Recommendations

1. Further research regarding the vertebra-pelvic ligament complex and its morphological variations is required
2. Additional clinical and radiological investigations are crucial in real patients with unspecified cause of lower back pain to explore the relation between idiopathic lower back pain and the variant type of the iliolumbar ligament
3. More studies are needed to investigate the etiology of lower back disc prolapse and if it has an association with the variant type of the iliolumbar ligament

## Figures and Tables

**Table 1:** The frequency of origins, insertions, number of bands, and accessory bands of the iliolumbar ligament.

Iliolumbar ligament			*N* = 20	%
Origin	Anterior part	TP of L5	14	70.0
		B of L4 and TP of L5	6	30.0
	Posterior part	TP of L5	16	80.0
		B of L5	4	20.0

Insertion	Anterior part	Iliac crest	20	100.0
	Posterior part	Iliac crest	20	100.0

Number of bands	Anterior part	1	1	5.0
		3	7	35.0
		4	4	20.0
		5	7	35.0
		6	1	5.0
	Posterior part	1	19	95.0
		2	1	5.0

Number of accessory bands	Anterior part	1	9	69.2
		2	4	30.8
	Posterior part	No	13	65.0
		Fused with anterior	7	35.0

**Table 2:** The distribution of length, width, thickness, and number of bands of the iliolumbar ligament.

Length of the anterior part	Minimum-maximum	1.22-5.99
	Mean	4.06
	SD	1.45

Length of the posterior part	Minimum-maximum	1.14-4.12
	Mean	2.44
	SD	0.82

Width of the anterior part	Minimum-maximum	0.62-2.60
	Mean	1.34
	SD	0.54

Thickness of the anterior part	Minimum-maximum	0.13-0.78
	Mean	0.41
	SD	0.21

Number of bands of the anterior part	Minimum-maximum	1.0-6.0
	Median	4.0
	IQR	3.0-5.0

Number of bands of the posterior part	Minimum-maximum	1.0-2.0
	Median	1.0
	IQR	1.0-1.0

## Data Availability

The cadaveric data used to support the findings of this study are included within the article.
